# CuO nanoparticles mixed with activated BC extracted from algae as promising material for supercapacitor electrodes

**DOI:** 10.1038/s41598-023-49760-4

**Published:** 2023-12-15

**Authors:** Marwah Ahmed Alsharif, Aishah Alatawi, Taymour A. Hamdalla, S. Alfadhli, A. A. A. Darwish

**Affiliations:** 1 Department of Physics, Faculty of Science, University of Tabuk, 71491 Tabuk, Saudi Arabia; 2 Department of Biology, Faculty of Science, University of Tabuk, 71491 Tabuk, Saudi Arabia; 3 Physics Department, Faculty of Science, Alexandria University, Alexandria, Egypt

**Keywords:** Environmental sciences, Materials science, Nanoscience and technology, Physics

## Abstract

The present analysis aims to use existing resources to lower the cost of electrodes and reduce environmental pollution by utilizing waste materials like green algae. In the present research, the hydrothermal carbonization technique was utilized to synthesize a nano sized CuO mixed with activated biochar (CuO@BC) extracted from red sea algae (*Chlorophyta*). The CuO@BC sample was extensively examined using several advanced physical techniques, such as UV/Visible spectroscopy, FTIR, XED, HRTEM, SEM, EDX, BET, and TGA. The HRTEM indicated that the size of the particles is 32 nm with a larger surface area and without aggregations. The BET analysis of CuO@BC indicates that the material contains pores of a relatively large size and with a pore diameter of about 42.56 A°. The electrochemical analysis of CuO@BC modified glassy carbon electrode CuO@BC/GCE has been investigated using CV, GCD, and EIS techniques. This CuO@BC/GCE shows excellent electrochemical features that are significant for energy storage applications. The CuO@BC/GCE showed a specific capacitance of approximately 353 Fg^−1^ which is higher compared to individual materials. Overall, the research outcomes suggest that the CuO@BC/GCE shows potential for use in high-performance supercapacitors as energy storage systems that are eco-friendly and sustainable.

## Introduction

Supercapacitors are high-performance energy storage devices due to their long lifetime, short charging times, exceptional safety features, and high power densities^1^. As a result, there is a greater public interest in energy conversion and storage materials^2^. Supercapacitors can be categorized into two groups depending on their energy storage mechanism: pseudo capacitors and electrical double-layer capacitors (EDLCs)^3^. The electrode materials that are sources of carbon are preferred in most supercapacitors which include carbon fiber cloth, activated carbon (AC), carbon nanotube (CNTs), carbon aerogel, and graphene^4^.

Activated carbon is a carbon material that has received attention because of its low cost, abundant source, easy accessibility, and excellent electrochemical properties^5^. Its stable structure, high carbon content, sizable specific surface area, and high degree of microporosity have made it famous^6–8^. Many natural waste materials such as rice husks, bamboo, corncobs, algae, pinecones, lotus stems, fruit peels, banana peels, and potato peels are transformed into biochar, which provides a large surface area and an abundance of electroactive sites for redox reactions and charge accumulation. Doping heteroatoms add significant properties to the carbon, enabling additional pseudo-capacitance from redox reactions depending on the heteroatoms’ functional group^9^. Biochar plays an important role in energy storage, electrocatalytic properties, and supercapacitor applications. Due to its porous nature, biochar has gained significant attention in the field of energy applications. The incorporation of metal oxides further enhances its energy storage capabilities^10^. The distinguishing characteristics of CuO-doped activated biochar include its increased surface area, porosity, and modified surface chemistry due to the incorporation of CuO. Furthermore, CuO acts as a catalyst, promoting chemical reactions that can degrade or transform hazardous compounds, such as organic pollutants or dyes. Finally, the presence of CuO could enhance the biochar’s ability to facilitate these reactions, resulting in more efficient pollutant removal or remediation.

**Figure 1:**
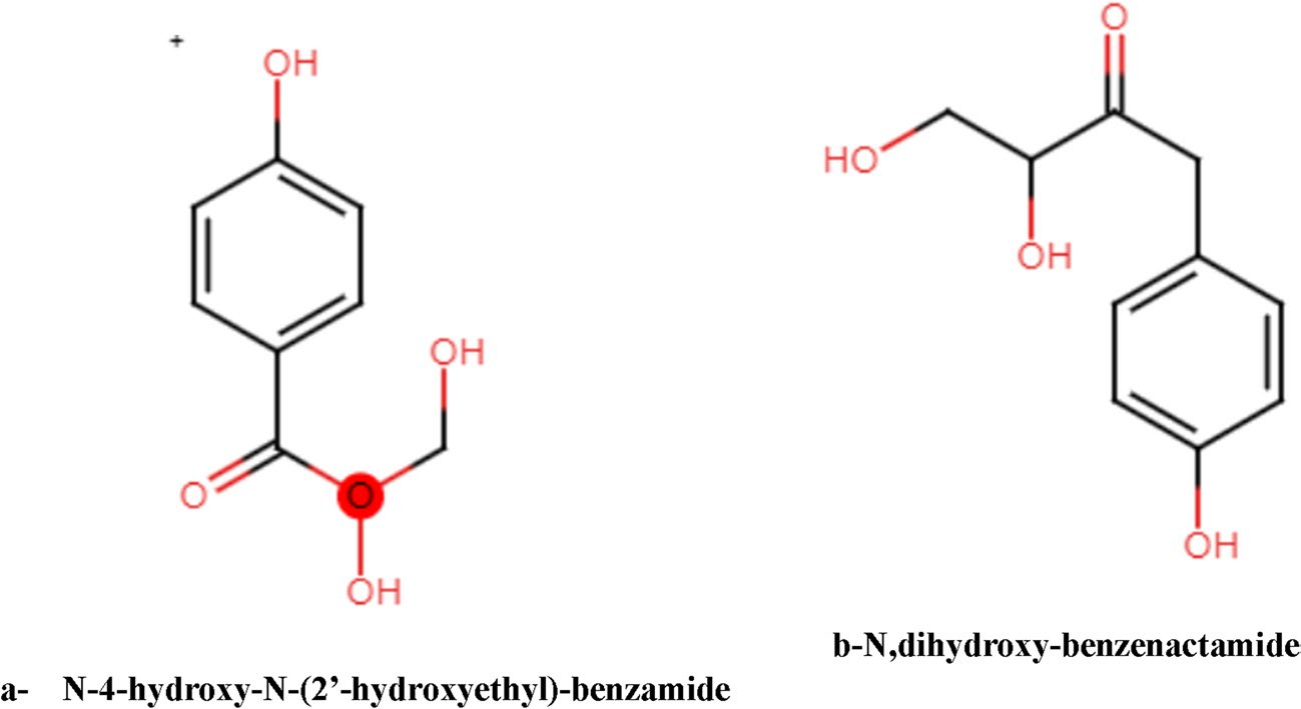
The chemical structure of the amide produced from Chlorophyta.

The green algae serve as a source of ecosystem services and biomass for various purposes such as food, nutraceuticals, soil additives, animal feed, and phycocolloids^11^. To utilize biomass waste and meet energy storage demands, converting such waste into biochar is an effective solution. Research has shown that biochar can act as an effective adsorption material due to its high surface-to-volume ratios, wide availability, sustainability^12^, and flaw sites. Biochars with high porosity can be obtained through physical/thermal and chemical activation (such as potassium hydroxide (KOH) and phosphoric acid (H_3_PO_4_)). Algae are a developing and sustainable source of biomass, with appreciable photosynthetic efficiency and less competition with food crops due to their requirement for a small land area. They are also known to detoxify the environment in food, agricultural feed, and other co-products^13^. The chemical structure of the amides produced from red sea algae is introduced in Fig. 1.

The Metal oxides and activated BC combination holds great potential for improving the performance and efficiency of supercapacitors, which are widely used for energy storage applications. Adding CuO to the electrode material enhances its capacitance, enabling higher energy and power densities. The biochar further contributes to this novel approach by providing a porous structure that facilitates ion movement and better electrochemical reactions. The synergistic effect of CuO and activated biochar creates an electrode with enhanced charge storage capacity and improved stability. By utilizing these materials, researchers strive to tackle the challenges associated with traditional supercapacitors, such as limited energy density and rapid self-discharge. This inventive collaboration between CuO@BC covers the way for enhanced supercapacitor technology, bringing us one step closer to efficient energy storage solutions for a sustainable future. Rai et al. investigated the electrochemical performance of pure CuO as an electrode material for energy storage^14^. They used cyclic voltammetry (CV) and galvanostatic charge–discharge (GCD) techniques to evaluate its performance. The results showed that CuO exhibited a capacity retention of 392.1 mAhg^−1^ which indicates its potential for energy storage applications. Dang et al. fabricated CuO nanoparticles with a unique hierarchical structure and evaluated their electrochemical properties^15^. The modified CuO nanosheets and nanorods demonstrated a significant improvement in specific capacity, achieving 600 mAhg^−1^. Furthermore, the modified CuO electrode exhibited excellent stability with minimal capacity fading during cycling.

**Figure 2:**
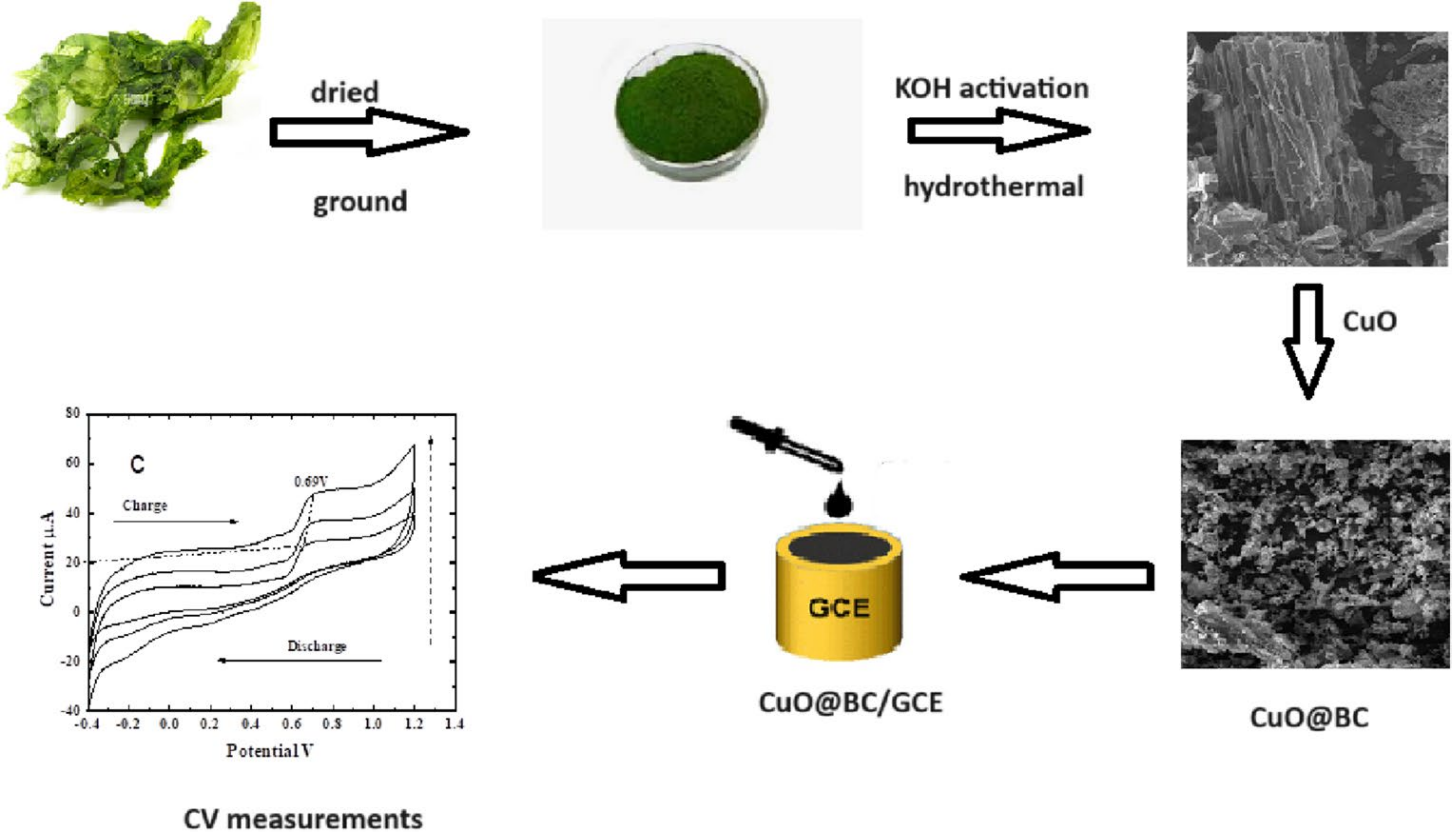
The experimental procedure of electrochemical measurements of CuO/BC.

To have an efficient energy storage electrode with a sustainable material, CuO NPs incorporated red sea algae (Chlorophyta) was successfully transformed into activated carbon using a hydrothermal method. The crystalline and morphological properties of CuO@BC composite have been done using various techniques such as UV/Visible, FT-IR, XRD, SEM, EDX, TGA, HRTEM, and BET. Then, the composite has been investigated as a potential electrode material for supercapacitor application. Figure 2 shows the experimental protocol of our thesis. Overall, incorporating biochar from algae with CuO in supercapacitors is a promising approach to creating environmentally friendly, cost-effective, and efficient energy storage devices with high performance.

## Materials and methods

### Preparation of activated carbon from Red Sea Algae biochar (BC)

The red sea algae were collected from the NEOM coast of Saudi Arabia. Chemicals such as copper sulfate (CuSo_4_ 98%), Ammonium hydroxide NH_4_OH (40%), sulfuric acid (H_2_SO_4_ 98%), potassium hydroxide (KOH 85%), *N*-methyl pyrrolidine (NMP), and dimethylformamide (DMF) were purchased from Sigma-Aldrich. The algae were washed with distilled water and dried at room temperature. Once dry, the algae were ground into a fine powder. The powder was pyrolyzed in a tube furnace for 4 h to prepare biochar. The flow rate of nitrogen gas was set to 50 mL per minute and the temperature of the furnace was maintained at 400 °C^16^. The first step involved adding 10 g of green algae powder to 100 mL of 5 M KOH in a reflux setup. The mixture was refluxed for 5 h at a temperature of 80 °C and with a stirring speed of 150 rpm. The second step required drying the residue from the first step and pyrolyzing it in the tube furnace under a nitrogen gas flow (700 °C, 4 h). Finally, the carbon material (BC) was washed with a solution mixture of Nitric acid (HNO_3_) and Sulfuric acid (H_2_SO_4_) (3:1). The material was then left to dry overnight at 60 °C to reduce impurities from the red algae powder.

### Preparation of Cu-modified biochar composite

The hydrothermal method was used to synthesize a Cu-modified biochar composite^17^. Prior to synthesis, 1 g of BC materials was added to 20 mL of Milli-Q water and the pH value was adjusted at 11 through CuSO_4_ and NH_4_OH. 4.75 g of biochar is mixed with 0.25 g of CuO so that the biochar: metal has a ratio of 100:5. The solution was then heated to 120 °C in a 33 mL Teflon-lined autoclave for 12 h. Centrifugation was carried out at 7000 rpm for 20 min, and the substance was purified using a 1:1 ethanol-Milli-Q water mixture at least three times. The final substance was then filtered and vacuum-dried at low temperatures.

### Preparation of CuO@BC NPs modified GCE

To investigate the electrochemical properties of CuO@BC composite, a glassy carbon electrode (GCE) was used. The GCE was cleaned by polishing it with alumina slurry, rinsing it with deionized water, and drying it with nitrogen gas. The GC electrode was de-coated with CuO@BC in the colloidal NPs solution at room temperature for the 2 h absorption time. A typical electrochemical system (CS-300 &150) was utilized to examine electrochemical measurements. The three-electrode system consisted of a counter electrode made of platinum (Pt), a working electrode modified with glassy carbon (GC), and a saturated calomel electrode (SCE) serving as a reference. The CV analysis of the GC electrode was studied in the presence of a potassium hydroxide (KOH 6 M) solution as the electrolyte, with a potential range of − 0.4 to 1.4 V and a scan rate of 50 mVs^−1^ at room temperature. The advantage of using alkali KOH electrolytes, compared to other organic electrolytes, is due to their higher concentration of ions and lower resistance for facilitating faster electrode kinetics. The CuO@BC/GCE was placed at room temperature to dry or placed in an oven for a specific amount of time to evaporate the solvent and ensure the adhesion of the nanocomposite to the surface of the electrode. To perform the electrochemical impedance spectroscopy (EIS) test, an amplitude of 5 mV and a frequency range of 0–2000 Hz were used.

## Results and discussion

### UV–visible spectroscopy

**Figure 3:**
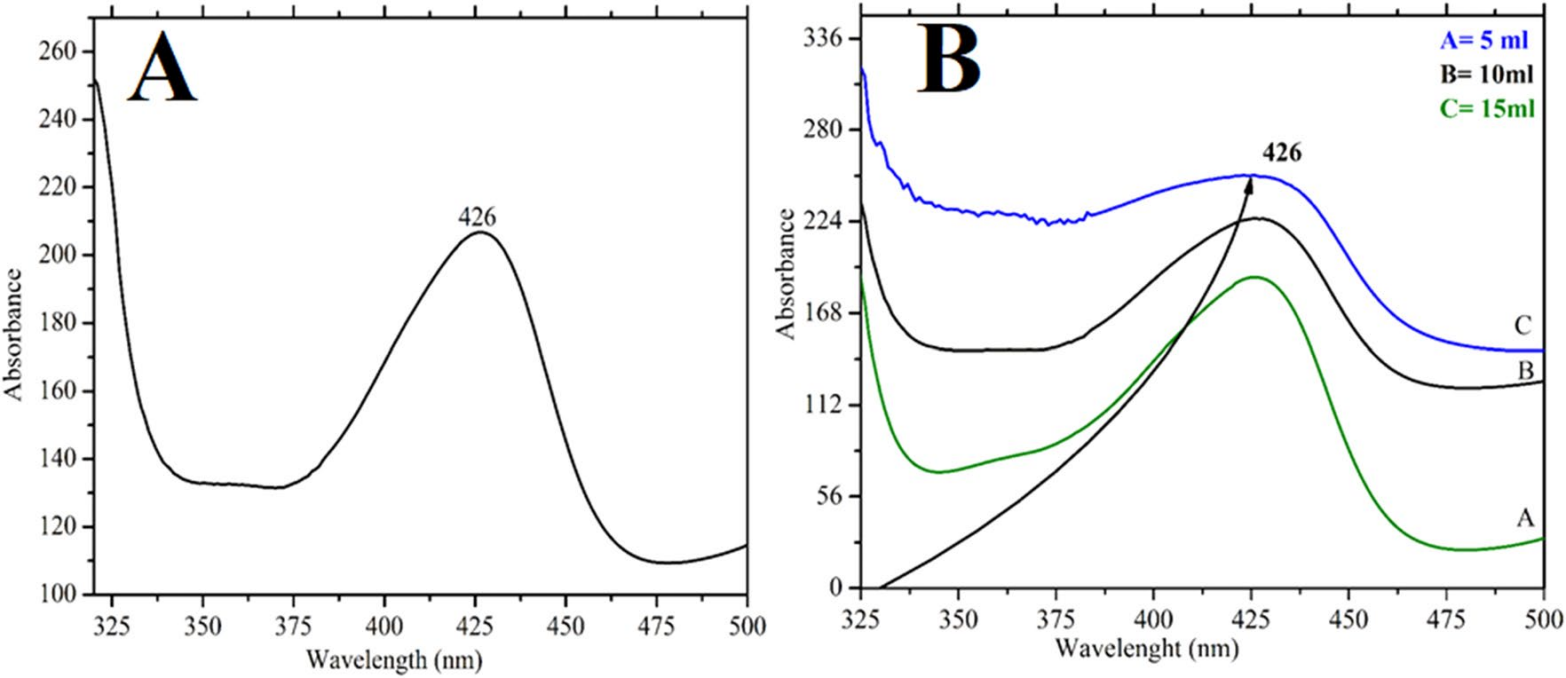
(**A**) UV–visible spectra of CuO@BC (**B**) effect of plant extract at the synthesis of CuO@BC.

UV/vis spectrophotometer was used to identify the wavelength of CuO@BC NPs as shown in Fig. 3. The plasmonic peak of the CuO was observed at 426 nm. UV/Vis spectroscopy excites valence electrons from HOMO to LUMO, which then shifts from a lower energy level to an energy level at a higher state (n–σ*, σ–σ*, n–π* and π–π*); this is determined by the ultraviolet spectroscopy & absorption helps to measure the gap in the energy created during this shift. The color change observed during exposure to plant extract indicates the reduction of Cu^+2^ into Cu0 nanoparticles. The reason behind this color change was the occurrence of Surface Plasmon resonance (SPR). Free electrons in metal nanoparticles give SPR absorption band at 426 nm. The SPR in the metal nanoparticles was excited, which caused the shading variations. The CuO@BC nanoparticles, which underwent photosynthesis, were periodically monitored using the scanning method during analysis of the sub-sample in the PerkinElmer Lambda 25 spectrometer, equipped with a UV–visible spectrometer. Typically, the conducting electron oscillates at specific wavelength ranges because of the SPR peak shown in Fig. 3A,B. The bioactive compounds and hydroxyl affect the particle size and shape of the CuO@BC synthesis and cause Cu reduction leading to metallic ions reduction^18^.

### FT-IR analysis

**Figure 4:**
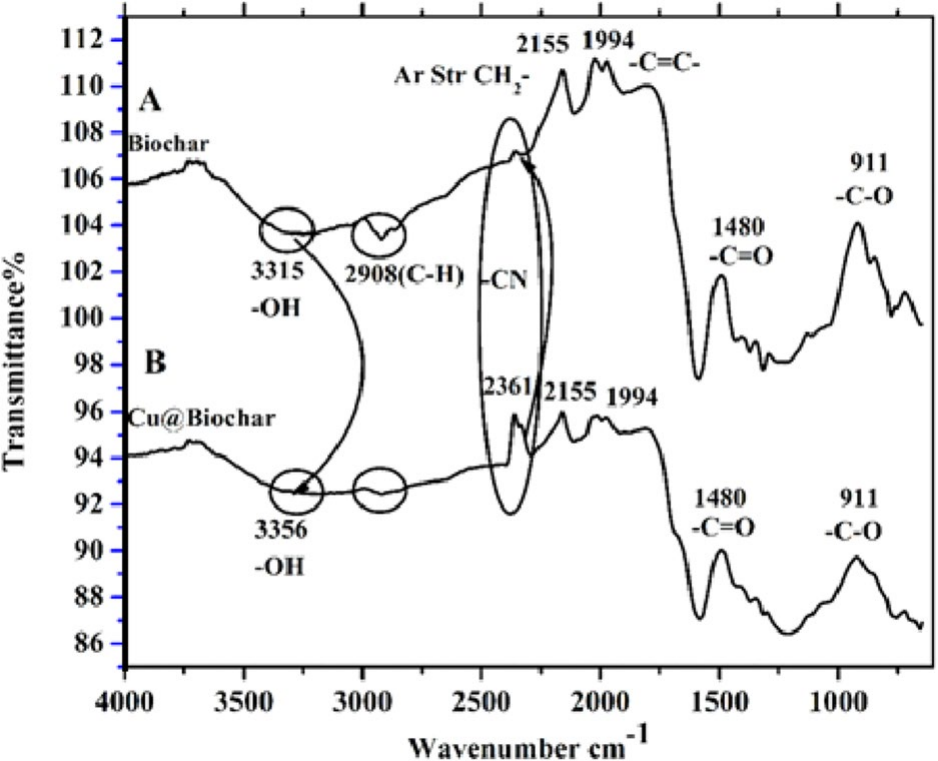
FT-IR (**A**) Biochar (**B**) CuO@BC NPs.

The Fourier Transform Infrared Spectroscopy (FTIR) spectrum is helpful in evaluating the vibrations of atoms, the structure of compounds, and the identification of functional groups^19^. Figure 4 shows the FTIR spectra in the wavenumber region 4000–400 cm^−1^ for BC and CuO@BC NPs. The peak at 3380 cm^−1^ confirms the alcoholic or phenolic character of the plant extract, indicating the stretching vibration of O–H bonds. The peak between 2739 and 2379 cm^−1^ is due to C–H stretching vibration. The peak at 1760 cm^−1^ is associated with the expanding vibration of keto and carboxylic groups’ C=O. The peak at 1596 cm^−1^ corresponds to the bending motion of the –C–O–H bond and the extension of the C–C bond. The peaks at 1316 and 1030 cm^−1^ indicate the bending vibrations of –C–H and –C–O–C. The peaks at 806 and 717 cm^−1^ confirm the production of H–CO and H–C=C. The reduction in peak intensities, suggests that the activated biochar affects the stability of nanomaterials.

### XRD analysis

**Figure 5:**
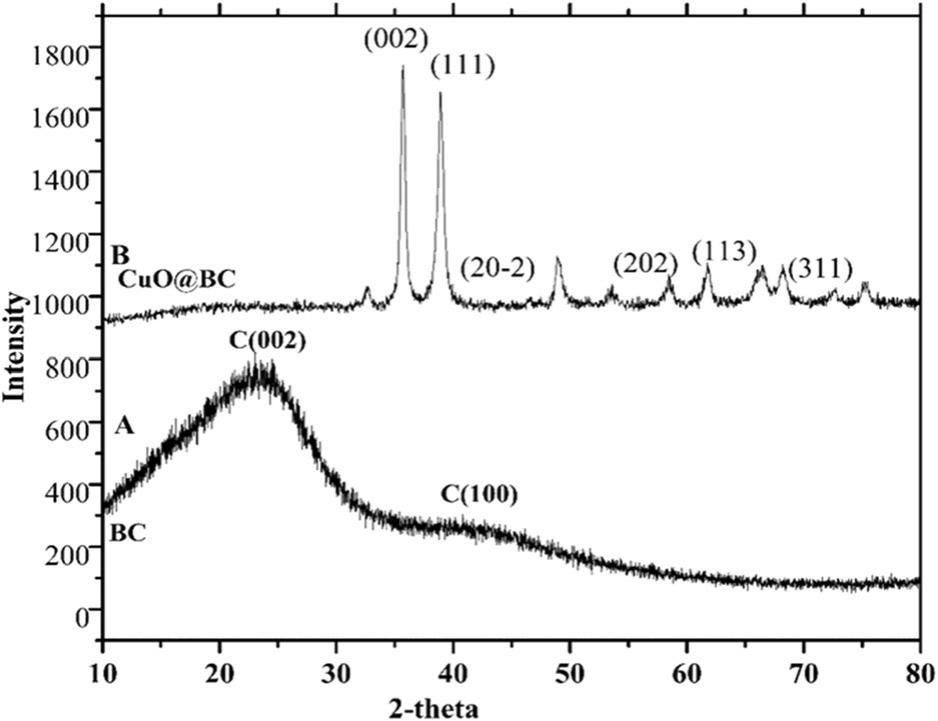
XRD of (**A**) BC (**B**) CuO@BC NPs.

The X-ray diffraction (XRD) technique is broadly used to examine the structural properties of particles; hence it helped define the crystalline structure of CuO@BC NPs, as shown in Fig. 5. X-ray diffraction analysis at 30–70° values confirmed that CuO@BC NPs were crystalline. To determine the value of the various Bragg reflection numbers at two thetas whose values are 36°, 39°, 59°, 62°, and 96°, correspond to 002, 111, 202, 113, and 311 set the location in lattice planes, respectively. The spectrum peak intensity for the 002 plane in the lattice structures suggests that CuO@BC has experienced greater expansion compared to the other patterns^20^. If the remaining peaks are not assigned and if biogenic materials are covering the surface of the CuO@BC, the XRD pattern indicates that the CuO@BC NPs are highly pure. By applying the Debye–Scherrer Eq. (1) and assuming that the average particle size was 32 nm which was corroborated by the method of measuring the particle size from the HRTEM picture and by using the equation^21^.

1 $${\text{D}} = \, 0.94 \, \lambda \, /\beta \cos \theta$$

where D stands for the particle size, β is expressed as the full-width half maximum in radian and θ is the Bragg angle.

### SEM analysis

**Figure 6:**
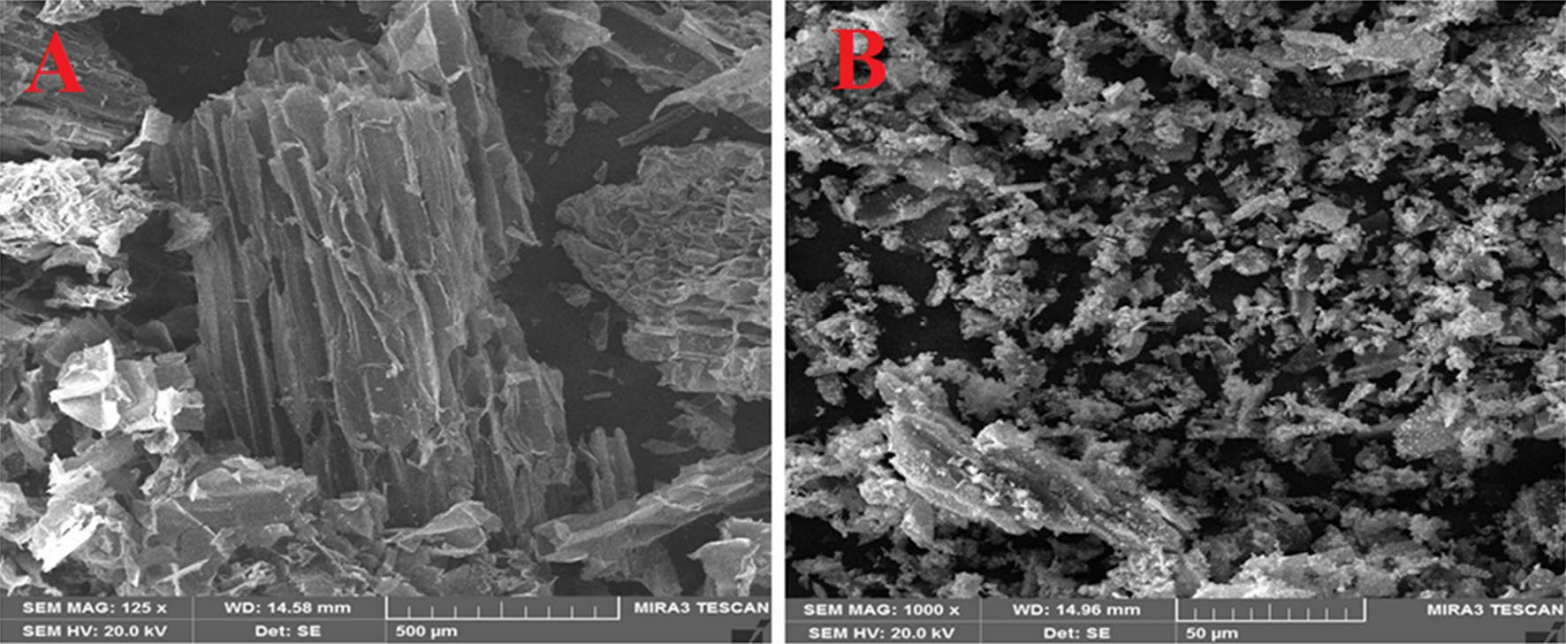
SEM images of (**A**) BC (**B**) CuO@BC NPs.

The surface activity of particles of the materials was usually studied by scanning electron microscopy (SEM) analysis^22^. This analytical method exposes the surface shape and particle size of the biogenic CuO@BC NPs. The SEM micrograph of BC is shown in Fig. 6A. As seen from the SEM images, BC has sheet-like structures appearing as stacked layers. Biochar structure could be helpful in improving the electrochemical properties that are accompanied by the increase in the surface area and porosity, which in turn provides a higher number of active sites for molecular interactions and charge transport. Furthermore, the stacked layers can create a unique and interconnected network for efficient charge transfer and ion mobility. Figure 6B shows the SEM image of CuO@BC NPs, the surface structure of CuO@BC NPs contains a combination of small and round particles related to CuO NP and loaded sheets. The surface morphology shaped a porosity in the material under investigation which can contribute to an increase in the material capacitance^23^.

### EDX analysis

Energy Dispersive X-ray (EDX) analysis of BC and CuO@BC NPs is presented in Table 1. EDX preparations showed the CuO reduction that influenced the crystalline nature of CuO@BC. The results show that there are Cu, C, and O present on the surface of the plant, alongside carbon and oxygen. The nutrients C and O come from plant extract^24^.

**Table 1:** EDX analysis for BC and CuO@BC.

Element	Line type	Weight %	Weight sigma	Atomic
BC				
C	K series	73.04	1.69	78.36
O	K series	26.96	1.69	21.70
Total		100		
CuO@BC				
CuO	K series	73.38	1.95	37.70
O	K series	14.84	1.11	30.28
C	K series	11.79	2.00	32.03
Total		100		100

### HRTEM analysis

**Figure 7:**
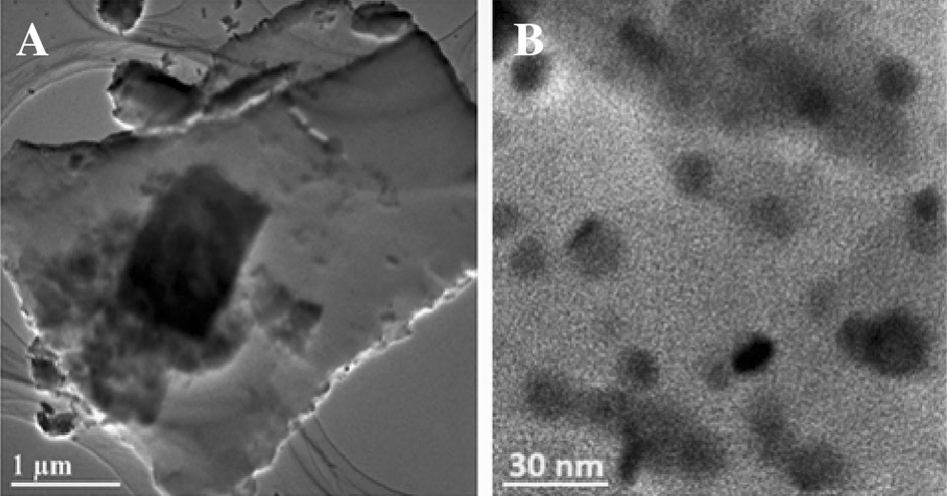
HRTEM images of CuO@BC NPs at (**A**) 1 µm, (**B**) 30 nm.

High-resolution transmission electron microscopy (HRTEM) can be an effective technique for investigating the size and shape of the CuO@BC. Due to its small and spherical shape, the surface area of the CuO@BC is very high and shows electrochemical and supercapacitor properties. HRTEM images of CuO@BC were shown with two different focusing on a material at a scale of 1 µm and 30 nm in Fig. 7. Detailed analysis exhibited that the composite possesses a spherical shape, with particles having a small size of approximately 32 nm. It is worth mentioning that spherical particles with nano-sized dimensions can result in a high specific surface area, providing more active sites for the electrochemical reactions in a supercapacitor, thus boosting its overall capacitance. Furthermore, it could lead to faster charge and discharge rates, as well as better overall conductivity^25^.

### TGA analysis

**Figure 8:**
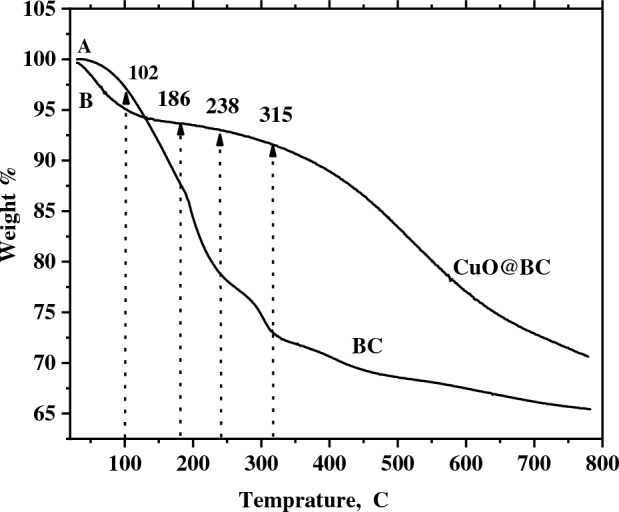
TGA spectra of BC and CuO@BC.

Thermal gravimetric analysis (TGA) can help to determine the thermal stability of the CuO@BC composite, which is important when assessing its suitability for supercapacitors. Information on the onset temperature of decomposition and the weight loss at various temperature ranges can help in modifying the composite’s stability for specific applications. The TGA of CuO@BC is shown in Fig. 8, and the loss of mass of CuO@BC NPs was determined at several phases of temperature and showed constancy up to 660 °C. As the temp. reaches 315 °C, the mass loss of BC and CuO@BC was about 22% and 7.5%, respectively. This suggests that CuO@BC has a high thermal stability up to 315 °C. The mass loss up to 315 °C indicates that there is likely some sort of decomposition or oxidation reaction occurring within the CuO@BC material. BC provides solid thermal stability to the green synthesized CuO@BC. Biochar’s porous structure can help to disperse heat better, reducing local temperature spikes and preventing rapid thermal degradation.

### BET analysis

Surface area analysis using a Nova 2200e surface area and pore analyzer (Quantichrome Instruments) was conducted. The sample was subjected to degassing at a temperature of 70 °C for a duration of 10 h before analysis. Nitrogen was utilized as an adsorptive gas at a temperature of 77 K. The BET surface analysis showed a value of 308 m^2^/g. The cumulative surface pore volume was measured to be 0.0819 mL/g, this value suggests an increase in the available pore space within the material. This can potentially enhance its adsorption capacity for various substances, such as heavy metals or organic pollutants. The pore diameter was found to be 42.56 A° which indicates that the material contains pores of a relatively large size. Such pores can allow for efficient mass transfer and provide accessibility for larger molecules. These results indicate that the biochar’s surface contains a significant amount of micropores.

### Electrochemical studies of CuO@BC

#### Cyclic voltammetry

**Figure 9:**
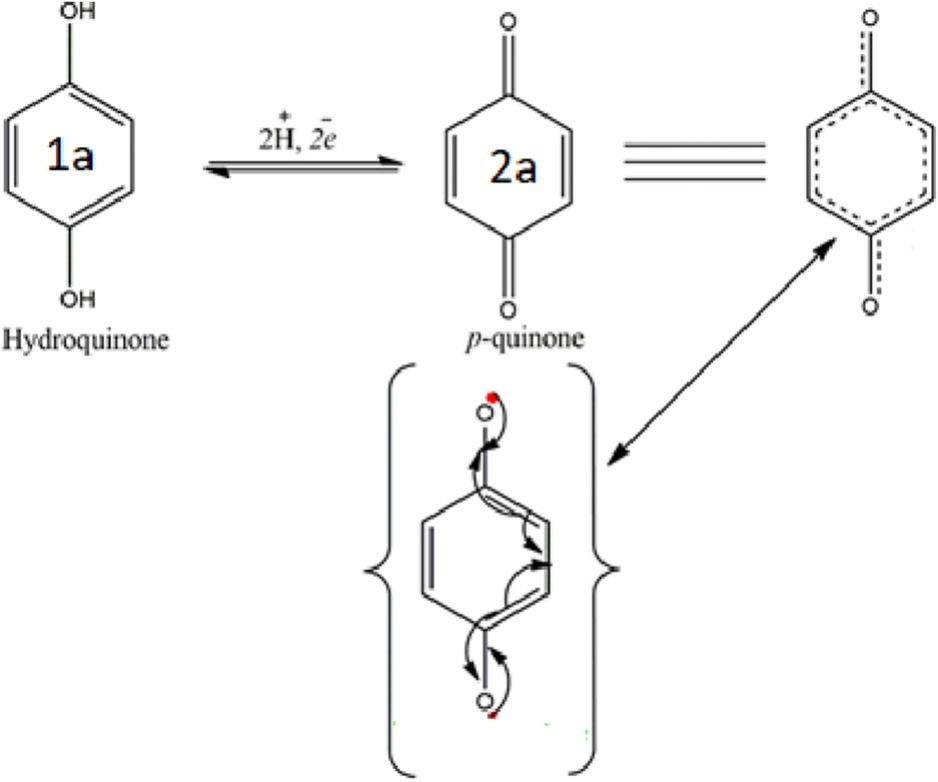
Electrochemical redox reaction of 1,4-dihydroxy benzene.

**Figure 10:**
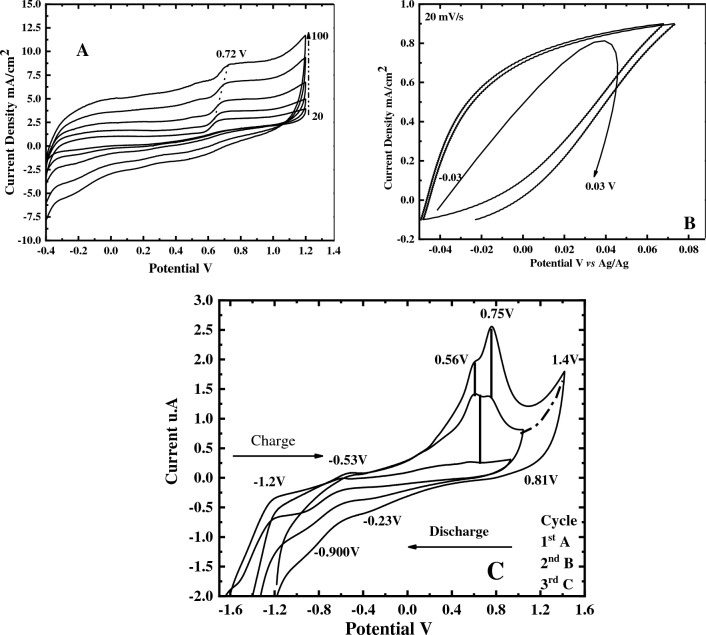
(**A**) CV curves of CuO@BC composite electrodes at 20–100 mVs^−1^ (**B**) curves CuO@BC composite at 1 A/g current density (**C**) Charge and Discharge of CuO@BC electrodes under − 1.6 to 1.6 V at 100 mVs^−1^.

The activated biochar material contains different types of functional groups, including –OH, –COOH, and other oxygen contents, that have a significant impact on the development of CV measurements^26^. When undergoing electrochemical oxidation, 1,2-dihydroxy benzene (catechol) (1a) is oxidized to create a highly reactive species quinone (2a) while showing the stability of (2a) at the electrode’s surface. Figure 9 shows the electrochemical redox reaction of 1,4-dihydroxy benzene. Figure 10A–C shows the CV curves of CuO@BC/GCE. Figure 10A shows the CV curves of CuO@BC composite electrodes at the scan rate of 20–100 mVs^−1^. All the curves of the modified electrodes in Fig. 10A display a quasi-rectangular shape with redox peaks indicating the behavior of an electrical double-layer capacitor EDLC with speedy charging and discharging of the materials^27^. This composite’s superior capacitance capability generates higher current density and integration area, as indicated by the curve.

Figure 10B shows the charge–discharge curves of the synthesized materials at a current density of 1A/g in a potential window ranging from − 0.06 to 0.08 V. We can conclude from Fig. that as the strength of the electric field increases, the electric polarization of the nanocomposite also increases until it reaches its maximum value. This indicates that there is an increase in the storage of electric energy^28^. Figure 10C shows the charge and discharge for CuO@BC/GCE in the potential range from − 1.6 to 1.6 V for three different cycles. As seen from Fig., an asymmetrical distribution in this electrode was identified due to its enhancing faradic property seen in the redox peaks of the curve, making it useful for an excellent electrical double-layer capacitor (EDLC). The synthesis of CuO@BC/GCE improves its capacitance value due to the formation of a large surface area, along with the higher electron transport efficiency within CuO@BC/GCE^29^. The synthesized nanomaterial composite exhibits higher diffusion properties due to the lower semicircle, resulting in a sound diffusion observed in this CuO@BC/GCE compared to other substances.

#### Specific capacitance of the CuO@BC

**Figure 11:**
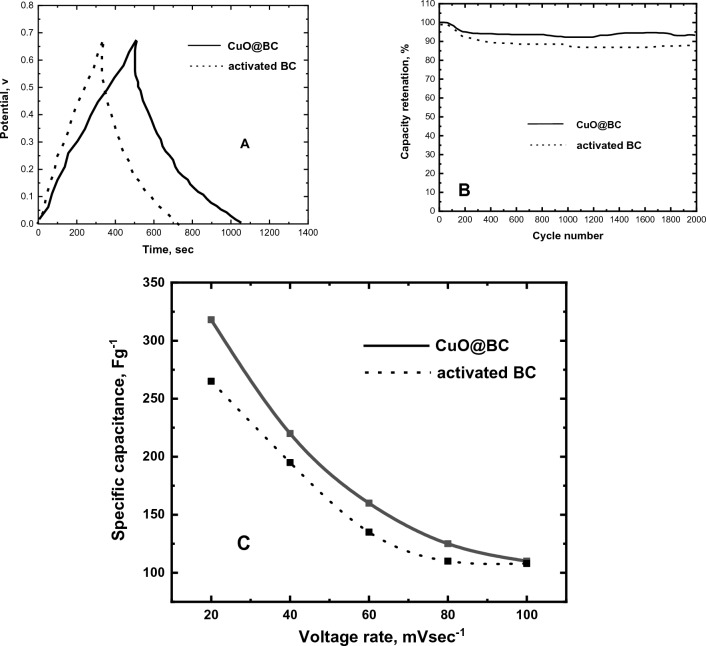
Supercapacitor charge–discharge characteristics and specific capacitance of activated BC and CuO@BC.

The capacitance of synthesized CuO@BC can be determined by utilizing^30^:

2 $$C = \frac{1}{{mv\left( {V_{c} - V_{a} } \right)\int\limits_{{V_{a} }}^{{V_{c} }} {I\left( V \right)dV} }}$$

where C is the capacitance in F/g, I is the current applied in A, ν is the potential rate (mV/s), V_c_ − V_a_ is the potential range, and m is load mass of CuO@BC composite in 1 cm^2^ deposited on the electrode that dipped in the electrolyte. The specific capacitance is calculated by^30^:

3 $$Cs = \frac{I\Delta t}{{m\Delta V}}$$

where *Cs* is the specific capacitance (F/g), ΔV is the potential, *I* is the discharge current in time (Δt), and m is the weight of deposited CuO material. Figure 11A shows the galvanostatic charge–discharge characteristics of activated BC/GCE and CuO@BC/GCE. The charge/discharge curves exhibited satisfactory symmetry with nearly triangular shapes for all current densities. This indicates that the material possesses commendable capacitive properties, even under high current density. A cycling test was conducted over 2000 cycles to investigate the stability of the CuO@BC/GCE, as depicted in Fig. 11B. As we can see, the capacity was retained at 94% after 2000 cycles for CuO@BC/GCE, this assures that our investigated electrode shows the stability during the CV cycling test. Figure 11C depicts the relation between the specific capacitance and scan rate for pure BC and CuO@BC/GCE. As current density increases, the capacitance decreases for activated BC and CuO@BC and this is due to electrolyte ions diffusing into the electrode material’s pores. The specific capacitance of CuO@BC obtained from the current measurements is introduced and compared with a previous study shown in Table 2.

**Table 2:** Comparison between the recent and other published electrochemical performance.

Precursors	Source biochar	Methods	Specific capacitance Fg^−1^	Ref.
Native sulfur/BC	Camellia japonica flowers	Chemical processs	125.42	^31^
NPCP/BC	Cornstalks	Thermochemical process	203.5	^30^
N/BC	Kapok (Ceiba insignis)	Microwave-assisted solvothermal	283	^32^
CuO	Algea	Hydrothermal carbonization method	353	Our work

#### Electrochemical impedance spectroscopy (EIS) of CuO@BC

**Figure 12:**
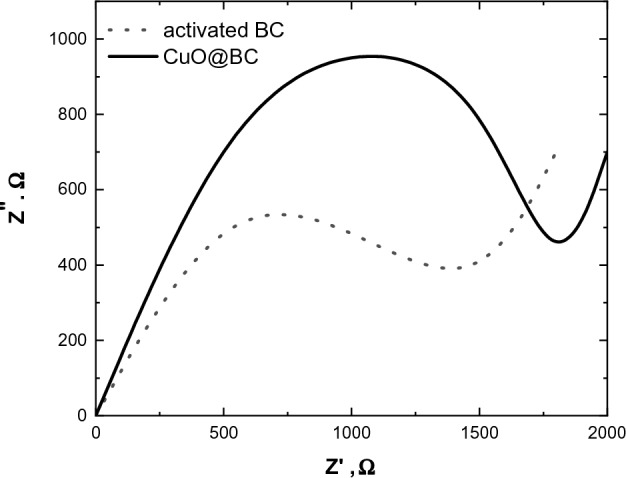
Impedance spectra of activated BC and CuO@BC.

Electrochemical Impedance Spectroscopy is a powerful and commonly used technique for characterizing the electrochemical properties of various materials, including composites such as CuO@BC. EIS of CuO@BC/GCE provides a valuable insight into several key parameters and properties, such as conductivity, surface properties, and electrochemical behavior, among others. By analyzing these properties, it is possible to optimize the composite’s performance for various applications, such as energy storage, environmental pollution remediation, and catalysis. The enhancement of the electrochemical performance of CuO@BC/GCE refers to the change in the complex impedance (Z* = Z′ + jZ″). Figure 12 shows the Z′ plotted against Z″ for activated BC and CuO@BC/GCE. As seen in the Fig., bulk resistance increases by incorporating the activated BC from 1400 to 1800 kΩ. Biochar is an organic compound primarily composed of carbon with relatively low electrical conductivity. When added to CuO, a semiconductor material with decent electrical conductivity, the overall conductivity of the resulting composite would decrease. This would lead to an increase in the bulk resistance of the CuO@BC/GCE compared to pure activated BC.

## Conclusion

In the present research work, the biochar obtained from red sea algae has been recognized as a valuable material for enhancing the performance of supercapacitors when combined with CuO NPs. The presence of CuO NPs in the biochar matrix affected the structure and morphology of BC. The electrochemical analysis ensured that the porous structure and high surface area of biochar added with CuO NPs contributed to increased capacitance by facilitating electrolyte diffusion, ion adsorption, and electron transfer. The CuO@BC electrode showed a specific capacitance of approximately 353 Fg^−1^ which is higher compared to individual materials. The EIS measurements of CuO@BC showed that the bulk resistance increases from 1400 to 1800 kΩ accompanied by the CuO addition in biochar. Consequently, the integration of CuO with biochar increases the specific capacitance making it an efficient and environmentally friendly option for the next generation of supercapacitors.

## Data Availability

The Authors confirm that the datasets generated during and analyzed during the current study are available from the corresponding author upon reasonable request.
